# Apoptotic Cell Clearance in *Drosophila melanogaster*

**DOI:** 10.3389/fimmu.2017.01881

**Published:** 2017-12-20

**Authors:** Qian Zheng, AiYing Ma, Lei Yuan, Ning Gao, Qi Feng, Nathalie C. Franc, Hui Xiao

**Affiliations:** ^1^Key Laboratory of the Ministry of Education for Medicinal Plant Resources and Natural Pharmaceutical Chemistry, National Engineering Laboratory for Resource Development of Endangered Crude Drugs in the Northwest of China, College of Life Sciences, Shaanxi Normal University Xi’an, Xi’an, China; ^2^College of Biological Sciences and Engineering, Beifang University of Nationalities, Yinchuan, NingXia, China; ^3^Department of Cell and Molecular Biology, The Scripps Research Institute, La Jolla, CA, United States

**Keywords:** phagocytosis, apoptosis, macrophages, signaling pathways, *Drosophila melanogaster*

## Abstract

The swift clearance of apoptotic cells (ACs) (efferocytosis) by phagocytes is a critical event during development of all multicellular organisms. It is achieved through phagocytosis by professional or amateur phagocytes. Failure in this process can lead to the development of inflammatory autoimmune or neurodegenerative diseases. AC clearance has been conserved throughout evolution, although many details in its mechanisms remain to be explored. It has been studied in the context of mammalian macrophages, and in the nematode *Caenorhabditis elegans*, which lacks “professional” phagocytes such as macrophages, but in which other cell types can engulf apoptotic corpses. In *Drosophila melanogaster*, ACs are engulfed by macrophages, glial, and epithelial cells. *Drosophila* macrophages perform similar functions to those of mammalian macrophages. They are professional phagocytes that participate in phagocytosis of ACs and pathogens. Study of AC clearance in *Drosophila* has identified some key elements, like the receptors Croquemort and Draper, promoting *Drosophila* as a suitable model to genetically dissect this process. In this review, we survey recent works of AC clearance pathways in *Drosophila*, and discuss the physiological outcomes and consequences of this process.

## Introduction

Programmed cell death is necessary for normal development and growth in multicellular organisms, which produce billions of apoptotic cells (ACs) daily (1, 2). Exogenous pathogenic microbes also threaten organisms’ lives and development (3). Swift and efficient removal of ACs and pathogens is essential for maintaining tissue homeostasis. Failure in this process results in the release of potentially cytotoxic or antigenic molecules, causing inflammatory diseases or developmental autoimmune disorders (4–7). To clear ACs and pathogens, multicellular organisms have evolved a conserved cellular process named phagocytosis that is being carried out either by non-professional or professional phagocytes (8). The molecular mechanism of ACs clearance has been extensively studied in *Caenorhabditis elegans*, thus revealing relatively clear and detailed engulfment pathways (9). However, *C. elegans* lacks the professional phagocytes; instead ACs are engulfed by many neighboring cell types (10). Absence of a professional immune system in *C. elegans* may limit the extent to which these data can be applied to higher organisms. The fruitfly *Drosophila melanogaster* has also been used as a suitable model to study ACs clearance, in which ACs are engulfed by both non-professional phagocytes such as epithelial cells and professional phagocytes such as macrophages/hemocytes and glial cells (11), providing the advantages for studying phagocytosis in mammals. ACs clearance proceeds when ACs expose “eat me” signals, which are recognized by phagocytes, thereby triggering signaling cascades that lead to internalization of the apoptotic corpse and its degradation by the phagocytic vacuole known as phagosome matures by fusing with lysosomes (12, 13). In this review, we will summarize the current research on phagocytosis of ACs in *D. melanogaster*, and which signaling pathways regulate this process, thereby giving a systematic and general overview of this process.

## Signaling by ACs in Phagocytosis

Apoptotic cells generated by programmed cell death or physical wounds are quickly and silently removed, to maintain tissue homeostasis or prevent auto-inflammatory responses (14, 15). Once cells begin to undergo apoptosis, cell death pathway is activated, and they release multiple signaling to recruit phagocytes, which contains three steps: the release of “find me” signals, the presentation of “eat me” signals, and the removal of “don’t eat me” signals (16).

At the beginning of cell death, “Find me” signals are released from ACs to promote the migration of phagocytes to ACs. Lauber firstly identified lysophosphatidylcholine (LPC) as a “find me” signal, which is released from ACs in a caspase-3-dependent manner. They furthermore showed that the activation of calcium-independent phospholipase A2 by caspase cleavage contributed to the release of LPC (17). Two other molecules, sphingosine-1-phosphate produced by sphingosine kinase in a caspase-dependent manner, and CX3CL1/fractalkine synthesized as a membrane-associated protein, have also been proposed to act as “find me” signals (18, 19). ATP and UTP that are released from ACs in a caspase-dependent manner have also recently been shown to act as “find me” signals for phagocytes (20). Whether these proposed “find me” signals are redundant or synergistic remains to be studied. Little evidence has shown that “find me” signals exist in *Drosophila*, but previous study revealed that H_2_O_2_ may be the immediate damage signal essential for the recruitment of hemocytes to wound regions in *Drosophila* embryos (21). Further research (22) found that Src42A–Draper–Shark signaling was important to recruitment of hemocytes by responding to wound-induced H_2_O_2_ in *Drosophila* embryos, which indicated that H_2_O_2_ may be *Drosophila* “find me” signal and Draper is responsible for the signal recognition. However, more evidence needs to be explored to verify this hypothesis.

“Don’t eat me” signals (also known as self-associated molecular patterns) exist on healthy cells, playing inhibitory roles to prevent to be engulfed by phagocytes. Some examples of “don’t eat me” signals include CD31, CD46, and CD47 in mammals (23).

“Eat me” signals are ligands, which can bind to engulfment receptors by moving to the surface of ACs. Engulfment receptors recognize and bind either directly to the apoptotic “eat me” signal, or through bridging molecules that bind the “eat me” signal. The best-studied and evolutionarily conserved “eat me” signal reported in human, *Drosophila*, and *C. elegans* is phosphatidylserine (PS) (24), a phospholipid exposed on the surface of ACs (25, 26). PS is a plasma membrane (PM) aminophospholipid maintained on the inner leaflet of live cells through aminophospholipid translocase activity (27, 28). After cell induced by apoptosis, aminophospholipid translocase is inactivated while a scramblase is activated to induce PS exposed to the cell surface in an ATP-independent manner (28). A recent study has shown that ACs can generate molecular memory in macrophages, priming them to recognize tissue wounds or microbes (29). This subsequently causes macrophages to produce pro-inflammatory signals and boost the innate response at sites associated with extensive AC death in *Drosophila* (29).

## Engulfment Receptors and Related Signal Pathways

In *Drosophila*, there are three cell types reported to function as phagocytic cells: professional phagocytes—macrophages/hemocytes, glial cells, and non-professional phagocytes—epithelial cells (30–32). Hemocytes are macrophage-like cells reported to engulf ACs or dendrite debris during pruning of *Drosophila* sensory dendrites (33) and embryogenesis (34). *Drosophila* glia act much similar role in engulfing dying cells or degenerating axons of the nervous system as their counterparts in mammals (35), degenerating dendrites are primarily cleared by the epidermal epithelia (36).

“Eat me” signals secreted by ACs are recognized by engulfment receptors, which are specifically expressed on the surface of phagocytic cells. In *C. elegans*, two seemingly independent engulfment signaling pathways have been genetically identified, which share similar functions both in fly and mouse, indicating that the process of ACs clearance is evolutionarily conserved. CED-1, a conserved transmembrane receptor protein, Draper in fly, MEGF10 in mouse, which have similar function in recognizing ACs, transducts the phagocytotic signal through its adaptor protein CED-6 (dCed-6 in fly, GULP in mouse) to regulate downstream effectors (37, 38). The CED-2, -5, -10, and -12 signaling pathway is believed to act downstream of the PS receptor PSR-1, a *C. elegans* homolog of mammalian PSR (39), which relates to ACs cytoskeletal rearrangements. Some of the abovementioned genes possess *Drosophila* counterparts, suggesting that fruitfly phagocytes share similar pathways to engulf ACs. Meanwhile, *Drosophila* has its own engulfment receptor, yet a more detailed mechanism remains to be unveiled in *Drosophila*.

### Croquemort

In 1996, Franc and colleagues cloned the first *Drosophila* engulfment receptor on embryonic macrophages, Croquemort (Crq), which shares 23% identity with human CD36. In mammals, CD36 act as a scavenger receptor engulfing ACs (40) and regulates the host inflammatory responses (41, 42). Crq expresses specifically on *Drosophila* plasmatocytes, which become macrophages as they encounter ACs from late stage 11 of embryogenesis (43). Using AC-labeling and Crq immunostaining experiments, Crq was shown to be required for efficient phagocytosis of ACs, which was also confirmed *in vivo* (34). Crq is structurally unrelated to either CED-1 or PSR-1 (34), and how it promotes phagocytosis, including the identity of its ligand, is still unknown (44).

In addition to macrophages clearing ACs during embryogenesis, epithelial cells are responsible for prompt clearance of degenerating neurites to maintain tissue homeostasis and prevent inflammatory responses during development (36). Knocking out *crq* results in AC clearance defects by macrophages; however, it has no effect on engulfment of dendrites in epithelia. Further studies showed that *crq* was required for phagosome maturation during this process, while loss-of-function of *crq* leads to homotypic phagosome fusion defect, though it is not necessary for phagosomes to progress through the Rab7^+^ positive stage (43). Besides, recent research revealed that *crq* mutant flies are susceptible to environmental microbes and infection, and that Crq is required for engulfment of bacteria in parallel to the Toll and Imd pathways, which play key roles in the innate immune system (45).

### Draper

Freeman and colleagues first identified the homolog of CED-1 in *Drosophila*, named Draper (Drpr), which strongly expressed on glial and macrophage membranes, and found that it was required for the engulfment of apoptotic neurons and for larval locomotion (35). Similar to CED-1 in nematode and MEGF10 in human, Drpr encodes 15 extracellular atypical EGF repeats, a single transmembrane domain, and a novel intracellular domain (35). Manaka and colleagues confirmed the role of Drpr in glia and hemocytes/macrophages, showing that it plays a role in the phagocytosis of ACs (44), suggesting that the Drpr pathway plays similar role in *Drosophila* as the ced-1/6/7 pathway in *C. elegans*. Glial cells expressing Drpr are essential for the pruning of *Drosophila* mushroom body γ neurons, Awasaki et al. detected that *Drosophila ced-6* (mouse *gulp*) expressed in the same glial cells as *drpr* (46), genetic evidence showed that *drpr* and *ced-6* played role in engulfing γ neuron axon in the same pathway, meanwhile, the experiment *in vitro* confirmed that Ced-6 N-terminal might interact with the intracellular region of Drpr (47). Different from *C. elegans* Ced-7, an ABC transporter, which both expresses in ACs and engulfment cells for efficient phagocytosis, the homolog in *Drosophila*, has not yet been studied. *Drosophila* Shark, a non-receptor tyrosine kinase also plays an important role in removing cell corpses or debris mediated by Drpr through binding to its intracellular domain (48). The Src family kinase Src42A phosphorylates Drpr to allow its intracellular domain to interact with Dmel/Ced-6, thus activating the Drpr pathway and promoting phagocytosis of pruned axons and degenerating neurons by glial cells (47).

In addition to the Ced-1, -6, and -7 signaling pathway, Ced-2, -5, -10, and -12 were found to act in a parallel and yet partially redundant pathway that controls actin cytoskeleton rearrangement in cell corpse engulfment and cell migration (49). For *Drosophila*, although the homologs of CED-2, -5, and -10 correspond to CG1587, myoblast city, and Rac2, respectively, their function in ACs clearance has not been deeply studied. The *Drosophila* homolog of Ced-12, Dmel/ced-12, was found to be required for cell clearance in macrophages, function in a genetically distinct pathway compared with Drpr, which further indicated that the phagocytosis signal pathways are evolutionary conserved (50).

### Integrin

Integrins are conserved heterodimeric transmembrane receptors, forming by two subunits called α and β (51, 52). The involvement of integrins in phagocytosis of ACs was first described in mammals (53). Ina-1, an α subunit of *C. elegans* integrin, was also reported to participate in cell corpse removal (54). In *Drosophila*, there are five α- and two β-subunits. Nagaosa and colleagues found that loss-of-function of *Drosophila* integrin *βv* results in reduced levels of AC clearance, while reexpressing *βv* in integrin *βv*-lacking fly hemocytes rescues their phagocytosis-defective phenotype (55). Flies lacking either integrin *βv* or Drpr showed almost the same level of phagocytosis, while loss of these two receptors further decreased phagocytosis, which indicated that integrin *βv* and Drpr act independently. As Drpr was shown to act upstream of CED-6 and CED-10, the integrin *βv* appears to act upstream of the other engulfment pathway CED-2–CED-5–CED-12. However, Crk and Mbc, the *Drosophila* homologs of *C. elegans* CED-2 and CED-5 have not been observed to participate in the phagocytosis of ACs at least by embryonic hemocytes, thus the molecular signaling downstream of *βv* remains unknown (55). Further research indicated that *Drosophila βv* acts as a phagocytic receptor to also promote clearance of *Staphylococcus aureus via* peptidoglycan binding on this bacterium (56). Another *Drosophila* integrin α-subunit, αPS3, also cooperates with *βv* in hemocytes and serves as an engulfment receptor for phagocytosis of ACs and *S. aureus* (57). In *Drosophila* ovary, highly polarized epithelial follicle cells (FCs) can engulf germline debris *via* their apical side. Meehan et al. (58) found that integrin heterodimer αPS3/βPS were apically enriched in engulfing FCs, which are required for engulfment of ACs by FCs. Thus, integrins are evolutionally conserved receptors that participate in AC clearance.

## Bridging Molecule

Several engulfment receptors have been identified that mediate phagocytosis of ACs, yet little is known about their precise mechanism of action, or whether they cooperate or act alone. Several molecules have been characterized that function upstream of Drpr to recognize ACs that are considered as “bridging molecules.”

### Six-Microns-Under (Simu)

Kurant and colleagues characterized a transmembrane protein named Simu, which is highly expressed on the surface of glial cells in the nervous system and macrophages elsewhere (59). Simu acts upstream of Drpr promote the recognition and engulfment of ACs (59). It strongly binds to ACs, through its EMILIN-like domain without membrane anchoring. Furthermore, Kurant and colleagues demonstrated that SIMU recognizes and binds PS secreted on ACs through its N-terminal EMILIN (EMI)-like domain, while the C-terminal NIM3 and NIM4 repeats regulate Simu affinity to PS (60). In addition, caspase activity is required for clearance of ACs by glial cells (60). However, the interaction mechanism between Simu and Drpr during clearance of ACs remains unclear, as Kurant and colleagues were failed to detect a directly physical linkage between Simu and Drpr (59). Thus, it seems likely that other molecules are required to connect these proteins (61).

### Calreticulin (Calr), Pretaporter (Prtp), and *Drosophila* Calcium-Binding Protein 1 (DmCaBP1)

Various proteins and lipids from the endoplasmic reticulum (ER) have also been found to be exposed at the surface of human ACs (62). Nakanishi and colleagues identified three ER proteins acting upstream of Drpr to promote phagocytosis in *Drosophila* (63–65). They showed that *Drosophila* Calr existed at the surface of living cells and reassigned to form aggregates upon apoptosis without change of the amount and expression at the cell surface; and that in a *Drosophila* mutant strain with reduced level of Calr, the level of phagocytosis of ACs was about a half of that observed in wild-type embryos (63). Thus, like PS, Calr is considered as a marker for phagocytosis of ACs in *Drosophila*. Through protein pull-down analysis, Nakanishi isolated an ER protein binding to the extracellular region of Drpr, with a signal peptide at the N-terminal and an ER retention motif at the C terminal, named Prtp. They found that Prpt relocated from ER to cell surface during apoptosis in *Drosophila* S2 cells (64), and they further showed that loss-of-function of *prtp* leads to reduced level of AC clearance both by embryonic hemocytes and embryonic glia. Reexpression of *prtp* in hemocytes did not rescue this defect while the ubiquitous expression did, which indicated that Prtp functions in ACs to promote phagocytes’ engulfment (64). The DmCaBP1 is released and externalized from ACs, to bind to the extracellular region of Drpr (65). Loss of either *prtp* or *DmCaBP1* led to a reduced level of AC clearance in *Drosophila* embryos, but the double mutant did not cause a further decreased in phagocytosis, which indicated that they act in the same pathway. As apoptosis induced, DmCaBP1 is externalized from ACs and serves as a bridging molecule to connect ACs and phagocytes, promoting efficient and timely phagocytosis to occur.

## E3, Ubiquitin Proteasome Pathway

By screening for genes required for efficient phagocytosis of ACs in *Drosophila* macrophages *in vivo*, Silva and colleagues identified *pallbearer* (*pall*), which encodes an F-box protein (66). F-box proteins are generally part of Skp/Cullin/F-box (SCF) complexes that act as E3 ligases targeting phosphorylated proteins to ubiquitylation and degradation *via* the 26S proteasome (67). In addition to F-box protein, the SCF complexes contain three constant polypeptides—Skp1, Cullin1 (Cul1), and Rbx1, which have their counterparts in *Drosophila*. In *Drosophila*, six Skp proteins have been identified; and only SkpA strongly expressed in the embryos (68), and Bocca reported that SkpA and Rbx1 interact with Lin19 (dCul1) respectively (69). Silva and colleagues showed that Pall physically interacts with SkpA *via* its F-box domain, the loss function of either Lin19 or SkpA resulted in phagocytosis-defective phenotype, which indicated that they constitute complexes to promote phagocytosis of ACs (66). Xiao and colleagues then identified one substrate of the Pall–SCF complex, namely, the ribosomal protein S6 (Rps6) (70). The F-box protein Pall interacts with phosphorylated Rps6, which induces its ubiquitination and degradation *via* the 26S proteasome pathway (70). As a consequence, Xiao and colleagues further showed that the Rac2 small GTPase was upregulated and activated, triggering actin cytoskeleton rearrangement and thus promoting the clearance of ACs (70). They also showed that Pall translocates from the nucleus to the cytoplasm upon AC exposure (70). However, the AC signal and molecular pathway that leads to Pall nuclear export has not yet been identified. Furthermore, the nature of the kinase that phosphorylates Rps6 upstream of its physical interaction with Pall and how the degradation of phosphorylated RpS6 results in higher levels and activation of Rac2 remain to be deciphered.

## Calcium Signaling

Calcium signaling is a second messenger, which participates in a number of cellular processes (71). Studies have identified several Ca^2+^ signaling genes that are required for AC removal in *Drosophila*. Cuttell and colleagues identified Undertaker (Uta) (also known as retinophilin), a *Drosophila* protein with membrane occupational recognition nexus repeats related to Junctophilin-like proteins, as required for Drpr-mediated phagocytosis (72). Junctophilins form junctional complexes between the PM and the ER or sarcoplasmic reticulum (SR) Ca^2+^ storage compartments that allow for cross talk between Ca^2+^ channels at the PM and the ER/SR Ca^2+^ channels (73). Cuttell and colleagues showed that the *Drosophila* ryanodine receptor, Rya-r44F, a Ca^2+^ channel on the ER membrane, also plays role in phagocytosis of ACs mediated by the Drpr pathway (71). They found that *uta* genetically interacts with *rya-r44F* upstream of the Drpr and Dmel/Ced-6 pathway to activate their downstream signaling cascade for efficient phagocytosis of ACs (72). Thus presumably, Uta forms junctional complexes between the PM and the ER to trigger the release of Ca^2+^ from the ER/SR compartment *via* Rya-R44F. Conversely, they showed that *drpr* and *Dmel/ced-6* are required for store-operated calcium entry (SOCE) *via* Stim and Orai (71). Thus, signaling downstream of Drpr and Dmel/Ced-6 may promote and/or maintain Uta-mediated junctional complexes, consequently mediating ER Ca^2+^ release to SOCE *via* Stim and Orai. It appears that Ca^2+^ functions in Drpr signaling downstream during both recognition and internalization of ACs, and Uta plays a central role both in Ca^2+^ homeostasis and phagocytosis. A similar link between Ca^2+^ homeostasis and AC clearance has been found in mammalian systems and *C. elegans* (74). Interestingly, a novel mechanism has been found by Weavers that *Drosophila* embryonic macrophages generate a memory after the uptake of ACs, priming them to detect tissue damage or infections. Engulfment of ACs associates with calcium bursts, increasing Drpr expression, which is important for the macrophages to rapidly respond and migrate to subsequent injury or infections (29).

## Cross Talk with Innate Immune Response

As phagocytosis is crucial for the normal development, it also plays important role in the immune response for the removal of ACs and pathogens (3, 24). The mechanisms that mediate phagocytosis of bacteria and how it interacts with other innate immune responses defense remain elusive. Hashimoto and colleagues showed that Drpr promotes phagocytosis of *S. aureus*, and *drpr* mutant flies show reduced resistance to a septic infection with *S. aureus* (75). *ltaS* encodes an enzyme responsible for the synthesis of lipoteichoic acid in *S. aureus* that acts as a ligand for Drpr in phagocytosis of *S. aureus* by *Drosophila* hemocytes. The integrin *βv* subunit promotes phagocytosis of *S. aureus* by binding to peptidoglycan of this bacterium (56), and the integrin αPS3 subunit cooperates with *βv* in this process (57). Guillou and colleagues showed that *crq* defective mutant flies appeared to be more susceptible to environmental microbes both during development and at adulthood, they further demonstrated that *crq* is required for microbial phagocytosis (45). Interestingly, AC clearance by *Drosophila* macrophages appears essential in priming these cells to respond to subsequent microbial infections *in vivo* (29). Macrophages that have not engulfed ACs fail to take up *E. coli*, while those that have previously engulfed ACs can recognize and take up *E. coli*, ultimately mediating the bacterium phagosomal degradation (29).

## JUN N-Terminal Kinase (JNK) Pathway

After recognition of ACs by macrophages and epithelial cells in mammals, the stress-activated MAP kinases JNK and p38 are activated at the early stage (76, 77). In *Drosophila* imaginal epithelia, normal imaginal cells exert an antitumor effect as oncogenic cells emerged to eliminate them (78). Ohsawa et al. revealed that the antitumor effect from surrounding cells was mediated by the activated JNK signaling, thus promoting the elimination of premalignant neighbors by engulfment (79). In *Drosophila* ovary, dying germline cells are cleared by neighbor follicular epithelia, which required Drpr signal pathway and activated JNK signal (80). During this process, Drpr acts upstream to activate JNK pathway, but another regulator exists to activate JNK pathway, which has not been studied. Their results suggested that the dying germline activates Drpr–JNK pathway, then JNK activity feeds back to increase Drpr expression in engulfing cells, which seem to be a circuit. Interestingly, although Ced-12 was showed to promote AC clearance in an independent pathway compared with Drpr, in *Drosophila* ovary, Timmons et al. (81) found that Ced-12 act upstream of JNK, which can to increase Drpr expression, similar to described earlier in *Drosophila* glia. As mentioned previously, glial cells play an important role in removing ACs during *Drosophila* embryonic development, neuronal pruning, and axonal degeneration (47, 59). Shklover showed that excess activation of JNK signaling in *Drosophila* embryonic glial cells does not affect the levels of Simu and Drpr expression but still promotes their apoptotic death and upregulates their phagocytic capacity by glial cells (82). As mentioned earlier, JNK signaling in follicular epithelia upregulates expression of Drpr, indicating that the phagocytosis induced by JNK signal may be tissue-specific. Recently, research showed that *Drosophila* glia upregulate their basal ability after neuronal injury, to phagocytosis through activation of the JNK pathway, which leads to the elevation of DRPR level (80, 83).

As mentioned previously, Weavers and colleagues proposed that ACs generate a molecular memory within macrophages, priming them to repair tissue damage and fight infection (29). They showed that JNK signaling is essential for macrophage detection of tissue damage and bacteria, as the uptake of ACs triggers calcium bursts in macrophages that induce JNK activation and signaling, ultimately leading to Drpr upregulation of expression (29).

## Conclusion

**Figure 1:**
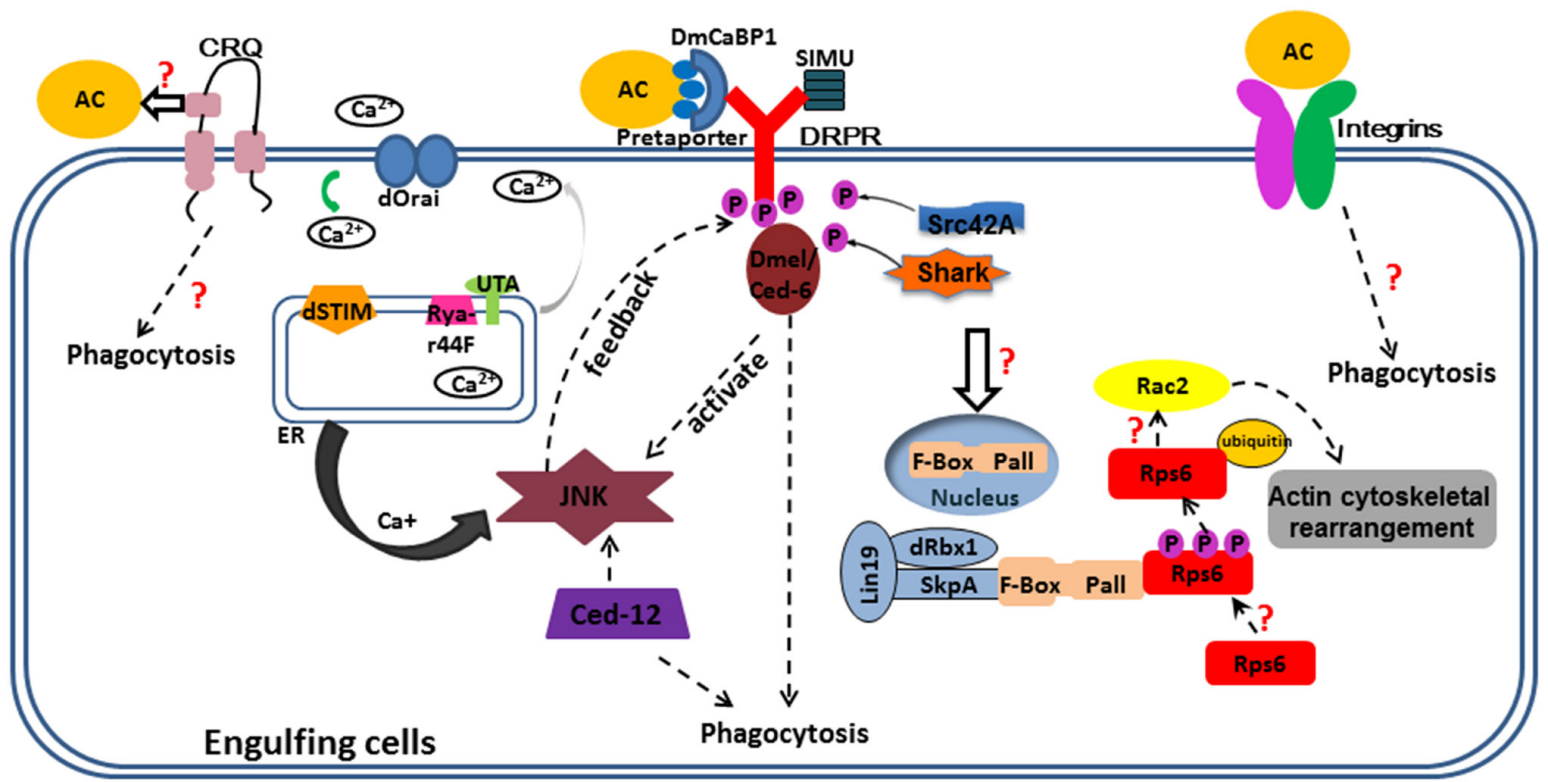
Overview of corpse recognition and disposal in *Drosophila*. Key components positioned according to their roles in corpse recognition, internalization, and processing. Some important questions are also indicated. Three engulfment receptors located at the plasma membrane. CRQ (Croquemort), a membrane two-path protein, expressed mainly in the macrophages and its ligand and downstream have not studied clearly; Draper (Ced-1), a membrane single-path protein, expressed mainly in glia of CNS and hemocytes, with its ligands are shown in this figure, phosphorylated Draper interact with Ced-6 thus elevates Jun N-terminal kinase (JNK) signal and maintain Ca^+^ homeostasis; JNK promotes Drpr enrichment both in glia and follicular epithelia (under Ced-12 activation). Integrins, a heterodimer protein, function in epithelial follicle cells and hemocytes to engulf cell corpses. The solid arrows mean the relationship between two proteins; the dotted arrows mean the molecular mechanism linking two pathways or proteins unknown. The purple circles with “P” in them mean phosphate groups. The orange circles with “AC” in them mean apoptotic cells; the rectangle outlined by blue lines means engulfment cells; different proteins or molecules are represented by colored shapes as shown in this figure.

Efficient and proper corpse clearance is important to maintain normal growth and prevent inappropriate inflammatory response, defective clearance of ACs often bring forth various diseases, such as autoimmune diseases, neurodegeneration, atherosclerosis, and Alzheimer’s disease. As AC clearance pathways were conserved from invertebrate to mammals, the typical pathways—*ced-1, -6, -7*, and *ced-2, -5, -10*, and *-12*, most-studied in worms or in mammals, also exist in flies. Over the past few decades, researchers have unveiled some of the molecular mechanisms of AC clearance in *Drosophila*. The process is outlined in Figure 1. However, multiple important questions concerning clearance mechanisms remain to be answered, and more detailed mechanisms remain to be explored. How does Crq recognize ACs and what is the ligand of Crq? Are there other engulfment receptors or regulators required for phagocytosis of ACs in *Drosophila*? How is Crq expression regulated by ACs? How does the translocation of Pall from nucleus to cytoplasm happen and which pathway regulates this event? Are the same regulatory mechanisms involved? What are the molecular mechanisms that mediate phagocytosis of bacteria and how do they overlap or differ from that of ACs? How does phagocytosis interact with other innate immune responses defense? To answer these questions, further studies of engulfment signals and the phagocytic machinery is required. In conclusion, our understanding of AC engulfment mechanism in *Drosophila* will enhance our theoretical foundation in this area, and provide a powerful complement to the research in mammals that could be useful for the development of therapeutic strategies to control diseases related to defective cell clearance.

## Author Contributions

QZ, AM, LY, NG, QF, NF, and HX wrote and reviewed the manuscript.

## Conflict of Interest Statement

The authors declare that the research was conducted in the absence of any commercial or financial relationships that could be construed as a potential conflict of interest. The reviewer KM declared a past coauthorship with the author NF to the handling editor.
